# Supplementary material for: Chemically mediated rheotaxis of endangered tri-spine horseshoe crab: potential dispersing mechanism to vegetated nursery habitats along the coast
Source: PeerJ. 2022 Dec 5;10:e14465. doi: 10.7717/peerj.14465 (PMC9745956; doi:10.7717/peerj.14465)
Supplement: Table S1 — Since the orientation data are not normally distributed, non-parametric Mann-Whitney U tests are adopted for the analyses. Conc., Concentration. [file peerj-10-14465-s002.docx]

**Table S1.**

Results of Student’s *t* tests (d.f. = 4) showing the effect of different inflow position of test waters (conditioned/control seawater), i.e., whether on the left or the right arm of the Y-maze chamber, on the behavioral responses of juvenile *T. tridentatus*. Since the orientation data are not normally distributed, non-parametric Mann-Whitney U tests are adopted for the analyses.

a) Conditioned waters

| **Habitat cue** | **Conc. (g l^-1^)** | **Orientation** | | **Movement time** | | **Largest distance** | | **Displacement** | |
| --- | --- | --- | --- | --- | --- | --- | --- | --- | --- |
|  |  | *z* | *p* | *t* | *p* | *t* | *p* | *t* | *p* |
| *A. marina* | 0.25 | -0.943 | 0.346 | 0.051 | 0.96 | -1.170 | 0.254 | -1.703 | 0.102 |
|  | 0.50 | -0.674 | 0.500 | -0.069 | 0.946 | 0.776 | 0.443 | 0.746 | 0.507 |
|  | 1.00 | -0.449 | 0.653 | 1.600 | 0.120 | -0.489 | 0.628 | -0.862 | 0.396 |
|  | 2.00 | -0.443 | 0.658 | -1.299 | 0.230 | -0.212 | 0.837 | 0.166 | 0.872 |
| *H. beccarii* | 0.25 | -0.899 | 0.369 | -0.87 | 0.391 | -1.01 | 0.321 | -1.397 | 0.173 |
|  | 0.50 | -0.674 | 0.500 | 0.707 | 0.483 | 0.547 | 0.587 | 1.865 | 0.069 |
|  | 1.00 | -1.328 | 0.184 | 1.044 | 0.302 | 1.459 | 0.151 | 0.899 | 0.373 |
|  | 2.00 | -0.674 | 0.500 | -1.064 | 0.296 | 1.685 | 0.102 | 0.779 | 0.442 |
| *S. alterniflora* | 0.25 | -0.674 | 0.500 | 0.443 | 0.662 | -0.346 | 0.732 | 0.596 | 0.557 |
|  | 0.50 | -0.221 | 0.825 | -1.994 | 0.059 | -0.536 | 0.597 | -0.303 | 0.765 |
|  | 1.00 | -0.886 | 0.376 | -0.007 | 0.995 | 0.372 | 0.712 | 0.327 | 0.746 |
|  | 2.00 | -0.696 | 0.487 | 0.163 | 0.873 | 0.460 | 0.653 | -0.271 | 0.791 |

Conc. = Concentration.

b) Control seawater

| **Habitat cue** | **Conc. (g l^-1^)** | **Orientation** | | **Movement time** | | **Largest distance** | | **Displacement** | |
| --- | --- | --- | --- | --- | --- | --- | --- | --- | --- |
|  |  | *z* | *p* | *t* | *p* | *t* | *p* | *t* | *p* |
| *A. marina* | 0.25 | 0.943 | 0.346 | -0.535 | 0.597 | -0.846 | 0.404 | -1.284 | 0.208 |
|  | 0.50 | 0.674 | 0.500 | 0.783 | 0.441 | -0.031 | 0.975 | 1.232 | 0.230 |
|  | 1.00 | 0.449 | 0.653 | -0.748 | 0.461 | -0.848 | 0.404 | 0.158 | 0.876 |
|  | 2.00 | 0.443 | 0.658 | 1.222 | 0.228 | 1.447 | 0.154 | 1.288 | 0.204 |
| *H. beccarii* | 0.25 | 0.899 | 0.369 | 0.241 | 0.811 | -0.708 | 0.485 | 0.043 | 0.966 |
|  | 0.50 | 0.674 | 0.500 | 1.410 | 0.180 | -0.671 | 0.513 | -0.573 | 0.576 |
|  | 1.00 | 1.328 | 0.184 | -1.701 | 0.123 | -1.087 | 0.305 | -1.593 | 0.146 |
|  | 2.00 | 0.674 | 0.500 | -0.096 | 0.924 | 1.165 | 0.255 | 1.093 | 0.284 |
| *S. alterniflora* | 0.25 | 0.674 | 0.500 | 1.595 | 0.120 | 0.791 | 0.434 | 0.835 | 0.410 |
|  | 0.50 | 0.221 | 0.825 | -0.260 | 0.795 | -0.278 | 0.783 | -1.616 | 0.115 |
|  | 1.00 | 0.886 | 0.376 | -1.723 | 0.098 | -1.601 | 0.123 | -1.791 | 0.086 |
|  | 2.00 | 0.696 | 0.487 | -0.373 | 0.711 | -1.652 | 0.106 | -1.396 | 0.170 |

Conc. = Concentration.
