# Supplementary material for: Chemically mediated rheotaxis of endangered tri-spine horseshoe crab: potential dispersing mechanism to vegetated nursery habitats along the coast
Source: PeerJ. 2022 Dec 5;10:e14465. doi: 10.7717/peerj.14465 (PMC9745956; doi:10.7717/peerj.14465)
Supplement: Table S2 — One sample was collected per site. [file peerj-10-14465-s003.docx]

**Table S2.**

Preliminary results on microalgae compositions and densities (10^5^ cells l^-1^) on the sediment surface of intertidal flats within Asian horseshoe crab nursery habitats along the northern Beibu Gulf shores, China. One sample was collected per site.

| **Microalgae composition** | | | | **Density** | |
| --- | --- | --- | --- | --- | --- |
| **Phylum** | **Class** | **Order** | **Family** | **Shanxin** | **Xibeiling** |
| Cyanophyta | Cyanophyceae | Chrococcales | Chrococcaceae | 2.42 | 4.33 |
|  |  | Oscillatoriales | Oscillatoriaceae | 1.27 | 4.42 |
| Bacillariophyta | Centricae | Coscinodiscales | Coscinodiscaceae | 0.63 | 1.40 |
|  | Pennatae | Araphidiales | Fragilariaceae | 1.53 | 1.66 |
|  |  | Aulonoraphididinales | Nitzschiaceae | 1.78 | 2.55 |
|  |  | Biraphidinales | Cymbellaceae | 6.75 | 7.64 |
|  |  |  | Gomphonemaceae | 1.02 | 3.18 |
|  |  |  | Naviculaceae | 9.43 | 10.70 |
|  |  | Monoraphidales | Achnanthaceae | 3.06 | 2.16 |
